# Disruption of PAK3 Signaling in Social Interaction Induced cFos Positive Cells Impairs Social Recognition Memory

**DOI:** 10.3390/cells10113010

**Published:** 2021-11-04

**Authors:** Susan Zhou, Zhengping Jia

**Affiliations:** 1 Department of Physiology, University of Toronto, Toronto, ON M5S 1A8, Canada; susan.zhou@sickkids.ca; 2 Department of Neuroscience and Mental Health, Hospital for Sick Children, Toronto, ON M5G 0A4, Canada

**Keywords:** p21-activated kinase, tetracycline-inducible system, cFos positive cells, social recognition memory

## Abstract

P21-activated kinase 3 (PAK3) gene mutations are linked to several neurodevelopmental disorders, but the underlying mechanisms remain unclear. In this study, we used a tetracycline-inducible system to control the expression of a mutant PAK3 (mPAK3) protein in immediate early gene, namely cFos, positive cells to disrupt PAK signaling, specifically in cells activated by social interaction in transgenic mice. We show that the expression of mPAK3-GFP proteins was in cFos-expressing excitatory and inhibitory neurons in various brain regions, such as the cortex and hippocampus, commonly activated during learning and memory. Basal expression of mPAK3-GFP proteins in cFos-positive cells resulted in social recognition memory deficits in the three-chamber social interaction test, without affecting locomotor activity or other forms of memory. The social memory deficit was rescued by doxycycline to halt the mPAK3-GFP transgene expression. In addition, we show that the expression of mPAK3-GFP proteins in a subset of cFos-positive cells, induced by an antecedent short social interaction, termed social pairing, was sufficient to impair social recognition memory. These results indicate that normal PAK signaling in cFos-positive cells activated during social interaction is critical for social memory.

## 1. Introduction

Social interactions are essential for forming meaningful relationships and for maintaining physical health [1,2,3], but unfortunately, many psychiatric and neurodevelopmental disorders, such as autism spectrum disorder (ASD), intellectual disability (ID), and schizophrenia, result in impaired social skills [4,5,6] that are currently without an effective treatment or cure. Non-syndromic sex-linked intellectual disability (NS-XLID), characterized by specific cognitive deficits that can affect social memory, without other major pathophysiology, such as altered brain development seen in syndromic ID, provides a unique venue for investigating the mechanisms underlying cognitive deficits [7,8]. NS-XLID can stem from gene mutations, among which the X-linked p21-activated kinase 3 (PAK3) is particularly prevalent. Genetic studies have shown that various gene mutations of PAK3 cause moderate to severe ID with abnormal dendritic development and synapse formation without affecting the gross brain development in human patients [9,10,11]. In addition, mouse models generated based on the manipulations of PAK3 and its close family members display impaired synaptic function, including spine morphology and synaptic plasticity, as well as cognitive deficits, without alterations in the gross anatomy of the brain, as seen in human patients [12,13,14,15,16,17]. Therefore, these animal models provide powerful tools for specifically investigating the molecular processes underlying cognitive deficits.

PAK3 is a serine-threonine protein kinase activated by Rho-GTPases, Cdc42, and Rac1 [18,19,20,21,22,23,24]. PAK3 can form homodimers or heterodimers with its close family member, PAK1, and therefore affects overall PAK signaling processes [25]. Previous in vitro and in vivo studies have indicated that PAK3 regulates spine morphogenesis, long-term potentiation (LTP), and spatial memory through the regulation of actin reorganization and gene expression [13,15,16,26,27,28]. Particularly, a recent study has revealed that PAK3 is important for social recognition memory in mice [17].

Learning and memory are believed to occur in a network of neurons referred to as engram cells [29,30]. During a learning experience, engram cells are activated and subsequently modified, and these modifications are thought to encode, store, and retrieve a memory [31,32,33]. The exact nature and cause of such modifications occurring in engram cells remain poorly understood. Recent studies have shown that synaptic changes, including LTP, are preferentially associated with engram cells, underscoring the critical importance of synaptic plasticity in engram cell modifications [34,35]. However, despite extensive studies of synaptic plasticity in the context of learning and memory, the molecular and cellular changes that are specifically linked to engram cells remain largely unknown.

Using the tetracycline-inducible system (tTA/tetO), we have previously shown that expression of a mutant PAK3 (R67)-GFP (mPAK3-GFP) protein in the excitatory neurons of the entorhinal–hippocampal (EC-HPC) circuit impaired social recognition memory without affecting other behaviors [17]. Therefore, PAK signaling in EC-HPC neurons represents a key molecular mechanism underlying social memory, but whether PAK signaling is required in engram cells remains unknown. In this study, we used the tTA/tetO system under the control of the cFos promotor to investigate the role of PAK signaling in neurons activated by social interaction. cFos is an immediate early gene (IEG) associated with cell activation during learning and subsequent long lasting synaptic plasticity, and therefore it is often used as a marker for engram cells [31,36,37,38]. We show that the inducible expression of mPAK3-GFP proteins in cFos-positive cells of adult mice was sufficient to impair social recognition memory. These results suggest that PAK-dependent molecular changes in engram cells are key mechanisms underlying social memory.

## 2. Materials and Methods

### 2.1. Animals and Doxycycline Administration

Animals were housed in a 12 h light/dark cycle (8 a.m./8 p.m.) and allowed ad libitum food and water, unless specified, with all experimental procedures done during the light cycle. Subjects were group-housed in cages of 2–5 same-sex animals and tested between 7 and 24 weeks of age. Both male and female animals were used. All experimental procedures were done in accordance with the guidelines of The Hospital for Sick Children and the Canadian Council on Animal Care.

The cFos-tTA/tetO-mPAK3-GFP double transgenic mouse line (dTg) was generated by crossing a cFos-tTA single transgenic (tTA-sTg) mouse line from Jackson Laboratory (018306 [39,40]) with a hemizygous tetO-mPAK3-GFP single transgenic (tetO-sTg) mouse line generated as previously described [17]. The genotypes of dTg mice and control sTg mice were confirmed through polymerase chain reaction for both the tTA (forward 5′-CGCTGTGGGGCATTTTACTTTAG-3′ and reverse 5′-CATGTCCAGATCGAAATCGTC-3′) and tetO-mPAK3 (forward 5′-GAACAGTAACAACCGAGACTC-3′ and reverse 5′-GGTGACTGCATCAAAACCCAC-3′) gene sequences, as previously described [13,17].

To turn off the mPAK3-GFP transgene expression, mice were treated with 50 mg/kg of doxycycline hyclate > 98% (DOX), administered in acidified drinking water for a minimum of 4 weeks, unless otherwise specified. To turn on the transgene expression, DOX was removed from the drinking water for 4 weeks, unless otherwise specified.

### 2.2. Behavioral Tests

All animals were handled twice daily for two days prior to behavior testing, and all experiments were performed with the experimenter blinded to the genotype of the subjects. All behavioral tests were recorded using either the ANY-Maze 6.1 (Stoelting Company, Wood Dale, IL, USA) or EthoVision software (Noldus Information Technology, Wageningen, the Netherlands), unless specified.

The open field and elevated plus maze tests were performed as previously described [17,41], where subjects were allowed to freely explore an open field apparatus (40 cm length × 40 cm width × 40 cm height) or a four-armed (closed arm is 40 cm length × 5 cm width × 10 cm height) elevated plus maze apparatus for 10 min.

A buried food test was performed, as described by others [42], where subjects were fasted for 18–22 h then given up to 10 min to find a food pellet buried 3 cm underneath the bedding. The novel object recognition test was performed as previously described [17,41]. Briefly, subjects were habituated for 5 min in the apparatus used for open field test, then introduced to two identical objects (O1a, O1b) during stage 1 for 5 min. Next, a novel object (O2) was introduced to replace either O1a or O1b during stage 2 for another 5 min, and finally another novel object (O3) replaced O2 during stage 3 for 5 min, where object interaction times were recorded in all stages.

The three-chamber social interaction test was performed as described before [17]. Subjects were habituated to the testing apparatus (20 cm length × 45 cm width × 30 cm height) and two empty stranger cages (E1, E2) 24 h prior, and an additional 10 min (stage 1) immediately prior to testing. Then, subjects were introduced to a sex-matched, juvenile stranger pup (S1) for 5 min (stage 2) and then either immediately introduced to a second novel sex-matched, juvenile stranger (S2) for 5 min (stage 3) or introduced to S2 24 h after stage 2 (stage 3—LTM), where all social interaction times were manually recorded. Social interaction was defined as any sniffing of the stranger initiated by the subject. The chambers that contained S1 and S2 were alternated for every other mouse to eliminate side preference bias. Similarly, the five-trial social interaction assay was performed in the center chamber of the same apparatus (20 cm length × 15 cm width × 30 cm height), where subjects were repeatedly introduced to S1 for 5 consecutive 1 min trials, with 30–45 s inter-trial intervals, and a novel S2 was introduced on the sixth 1 min trial [17].

In our social pairing protocol to activate potential engram cells, mice were first treated with DOX for 4 weeks to turn off the mPAK3-GFP transgene expression. Afterwards, subjects were single-housed and DOX was removed for 48 h. During the last 2 or 24 h of the 48 h window, a sex-matched juvenile stranger (S1) was introduced into the home cage and remained with the subject until the end of the 48 h window (social pairing). Subjects were all placed back on DOX after removal of S1 and a modified three-chamber social interaction test, with only stages 1 and 3, was performed 24 h after pairing to assess long-term social memory for the paired S1.

Our Morris Water Maze protocol was modified from previously described protocols [43,44], in which subjects were placed into a 130 cm diameter pool of room-temperature water, colored with non-toxic white paint, and allowed to swim freely for 60 s or until they located and stayed on the platform for 4 s; the training was repeated for 3 total sessions per day. Each session utilized a different drop-off point located in one of the three quadrants that did not contain the platform, and the order of drop-off points were alternated every day. Subjects were first trained for 2 days in a visible platform test with a flagged platform in the southwestern quadrant, followed by 11 days of training with a hidden platform in the northwestern quadrant, and finally 8 days of reversal platform training with a hidden platform in the southeastern quadrant. Hidden and reversal spatial memory was tested using probe trials lasting for 30 s, 2 and 24 h after the final training session, in which the platform was removed, and time spent in each quadrant zone was recorded. The time spent in the platform zone was compared against a combined average of the remaining zones.

Fear conditioning protocols were adapted and modified from previous studies, [44,45] in which subjects were habituated in a context A chamber (20 cm length × 21 cm width × 19 cm height) for 2 min, then two tone-shock sessions were administered with a 1 min inter-session interval. Each tone-shock session consisted of a 30 s 85 dB tone (CS) and mild 0.3 mA foot shock (US) during the last 2 s of the tone. Contextual fear memory was tested by placing the subject back into the context A chamber for 5 min without any tone or shock, and cued fear memory was tested by placing the subject into a novel and visually distinct context B chamber (20 cm length × 21 cm width × 19 cm height) for 5 min, with a similar timeline including two 30 s tone only sessions with 60 s inter-session intervals. Contextual and cued fear memories were tested 2 and 24 h after training, and the percentage of freezing was recorded and analyzed with FreezeFrame and FreezeView software (Actimetrics, Wilmette, IL, USA).

### 2.3. Immunohistochemistry and Fluorescent Imaging

Subjects were anesthetized with an intraperitoneal injection of 100 mg/kg ketamine hydrochloride and 10 mg/kg xylazine and were given a transcardial perfusion of 20–25 mL of 1X phosphate-buffered saline (PBS), followed by 20–25 mL of 4% paraformaldehyde (PFA). Brains were post-fixated in 4% PFA overnight at 4 °C, then washed with 1X PBS the next day and transferred to 30% sucrose for 24 h, or until the brain fully sank to the bottom of the container. Washing with PBS consisted of 3 separate washes, with each wash lasting for 5 min. Fixed brains were embedded and stored in Tissue-Tek OCT compound embedding medium at −80 °C until ready for cryosection into 25 μm coronal sections at –20 °C. Sections were collected on glass microscope slides and dried for at least 25 min in room temperature before storing in −20 °C until staining.

Brain sections were washed with PBS, permeabilized with 0.1% Triton X-100 in 1X PBS for 15 min, and washed with PBS before blocking with 5% BSA in 0.1% Triton X-100 in 1X PBS for 60 min at room temperature. Sections were washed with PBS, lined with a hydrophobic PAP pen and incubated with primary antibodies in 5% BSA in 1X PBS overnight at 4 °C. The next day, sections were washed with PBS, then incubated with corresponding secondary antibodies in 5% BSA in 1X PBS for 120–150 min in room temperature. Finally, the sections were washed once more with PBS before mounting with VectaShield hard-set anti-fade mounting media with DAPI and cover-slipped to dry for 60–120 min before imaging.

Immunohistochemical primary antibodies used included: 1:1000 chicken anti-GFP (GFP-1010, Aves Labs Inc., Davis, CA, USA), 1:500 rabbit anti-NeuN (12943, Cell Signaling Technology, Danvers, MA, USA), 1:500 mouse anti-GFAP (3670, Cell Signaling Technology), 1:1000 rabbit anti-cFos (2250, Cell Signaling Technology), 1:500 rabbit anti-CDP (sc-13024, Santa Cruz Biotechnology, Dallas, TX, USA), 1:200 mouse anti-GAD67 (sc-28376, Santa Cruz Biotechnology), and 1:1000 mouse anti-CaMKIIα (MA1-048, Thermo Fisher Scientific, Waltham, MA, USA). Secondary antibodies used included: 1:1000 Alexa Fluor 488 Donkey anti-Chicken IgY (703-545-155, Jackson ImmunoResearch Laboratories, West Grove, LA, USA), 1:1000 Alexa Fluor 546 Goat anti-Mouse IgG (A-11003, Invitrogen, Waltham, MA, USA), and 1:1000 Alexa Fluor 568 Donkey anti-Rabbit IgG (A10042, Invitrogen).

Images were taken with either the Leica DFC7000T microscope camera (Leica Microsystems, Wetzlar, Germany), Zeiss Axiocam 503 mono (Carl Zeiss, Jena, Germany), Zeiss Axiovert 200M (Carl Zeiss), or Zeiss LSM 880 with Airyscan (Carl Zeiss) with 10X or 20X lens objectives. Imaging parameters were kept consistent within genotypes of the same experiment during image acquisition. Cell quantification analyses were done through manual counting of colocalizing cells using the Fiji ImageJ 1.52n program (Laboratory for Optical and Computational Instrumentation, Madison, WI, USA).

### 2.4. Statistical Analyses

All statistical analyses were performed on GraphPad Prism 6 and 8 (GraphPad Software), and all data are represented as mean ± standard error of the mean (SEM). Behavioral data were tested with the Shapiro–Wilk test for normality, then two-tailed paired and unpaired *t*-test, repeated measures two-way ANOVA, and one-way ANOVA tests were applied where applicable. Data with significance were further analyzed with post hoc Holm–Sidak’s multiple comparisons. Significance is reported as * *p* ≤ 0.05, ** *p* ≤ 0.01, *** *p* ≤ 0.001, **** *p* < 0.0001, where *p* ≤ 0.05 is considered as significant.

## 3. Results

### 3.1. mPAK3-GFP Transgene Is Expressed in cFos-Expressing Excitatory and Inhibitory Neurons under Basal Conditions

**Figure 1:**
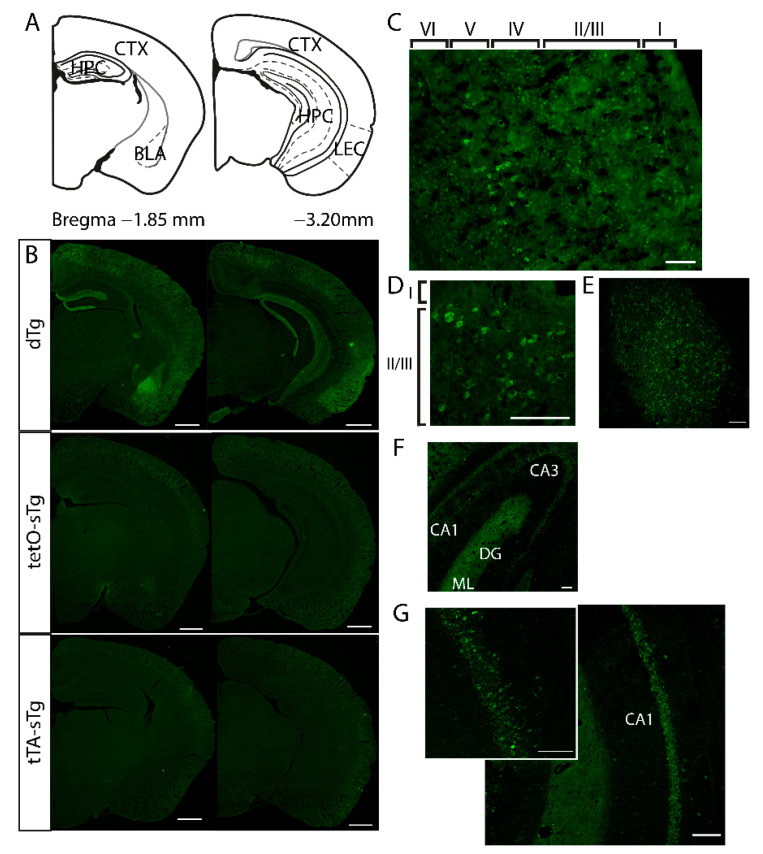
The basal expression of mPAK3-GFP proteins in sTg and dTg animals. (**A**) Schematic of the regions of interest in dorsal and posterior coronal sections. (**B**) The global expression of mPAK3-GFP proteins in dorsal and posterior coronal half brain sections from dTg and control, tetO-sTg, and tTA-sTg mice. Scale bar = 1000 μm. (**C**–**G**) Higher magnifications of the regions of interest in dTg mice. Expression of mPAK3-GFP proteins can be seen in (**C**) all cortical layers, including the (**D**) LEC, as well as the (**E**) BLA, and (**F**) hippocampal CA3 and (**G**) CA1 regions. Scale bars: 100 μm. CTX, cortex; HPC, hippocampus; BLA, basolateral amygdala; LEC, lateral entorhinal cortex; dTg, double transgenic; tetO-sTg, tetO-mPAK3-GFP single transgenic; tTA-sTg, Fos-tTA single transgenic; CA1, cornus ammonis 1; CA3, cornus ammonis 3; DG, dentate gyrus; ML, molecular layer; GCL, granule cell layer.

We first examined the expression patterns of mPAK3-GFP proteins in cFos-tTA/tetO-mPAK3-GFP dTg mice under basal conditions without any learning experience by immunostaining for anti-GFP in coronal brain sections. We found that in contrast with single transgenic mice (i.e., tTA-sTg containing the cFos-tTA transgene only or tetO-sTg containing the tetO-mPAK3-GFP transgene only), which showed no expression of mPAK3-GFP proteins, dTg animals expressed mPAK3-GFP proteins in the cortex, dorsal and ventral HPC, and basolateral amygdala (Figure 1A–G). Within the HPC, dTg animals showed higher levels of mPAK3-GFP proteins in the CA1, CA3, and the dentate gyrus molecular layer (DG ML) (Figure 1F,G).

**Figure 2:**
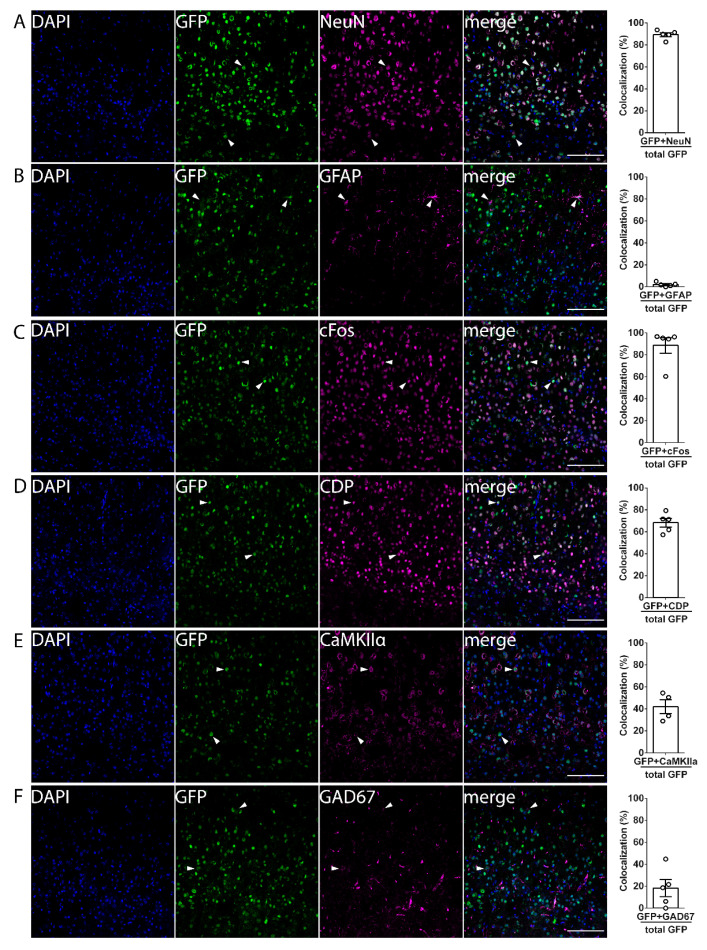
The colocalization patterns of mPAK3-GFP proteins in the cortical layers of dTg animals. (**A**–**E**) The individual and merged immunostaining images of DAPI (blue), GFP (green), and RFP (magenta) in coronal cortical sections of adult dTg mice. Cells expressing mPAK3-GFP proteins in the cortex colocalized with (**A**) neuronal NeuN (89.41 ± 1.831%), (**C**) immediate-early gene cFos (88.64 ± 7.073%), (**D**) layer 2/3 CDP (68.32 ± 3.910%), (**E**) excitatory neuronal CaMKIIα (41.85 ± 6.265%), and (**F**) inhibitory GAD67 (18.21 ± 7.718%), but rarely with (**B**) astrocytic GFAP (2.065 ± 0.8473%). Percentages represent cells with colocalization over total mPAK3-GFP cells. Scale bars = 100 μm. NeuN *n* = 5, GFAP *n* = 5, cFos *n* = 5, CDP *n* = 5, CaMKIIα *n* = 4, GAD67 *n* = 5. White arrows indicate sample mPAK3-GFP-expressing cells with and without colocalization in all images. dTg, double transgenic; GFP, green-fluorescent protein; RFP, red-fluorescent protein; DAPI, 4′,6-diamidino-2-phenylindole; GFAP, glial fibrillary acidic protein; CDP, CCAAT-displacement protein; CaMKIIα, calcium/calmodulin-dependent protein kinase II alpha; GAD67, glutamate decarboxylase 67. Data are expressed as mean ± SEM. *n* = number of mice.

Next, to determine the cell types expressing mPAK3-GFP proteins, we performed colocalization staining using anti-GFP, together with either anti-NeuN, anti-GFAP, anti-cFos, anti-CDP, anti-CaMKIIα, or anti-GAD67 to identify neurons, astrocytes, cFos protein expressing cells, cortical layer II/III (L2/3) cells, CaMKIIα-expressing excitatory cells, or inhibitory cells, respectively (Figure 2). We found that mPAK3-GFP-expressing cells in dTg animals co-localized mostly with neurons (Figure 2A, 89.41 ± 1.831%) and cFos positive cells (Figure 2C, 88.64 ± 7.073%), and with a subset of excitatory neurons (Figure 2D, CDP 68.32 ± 3.910%, E, CaMKIIα 41.85 ± 6.265%), and a smaller proportion of inhibitory neurons (Figure 2F, 18.21 ± 7.718%), but minimally with astrocytes (Figure 2B, 2.065 ± 0.8473%). These results indicate that mPAK3-GFP proteins are mainly expressed in cFos-positive neurons under home-cage housing conditions, when not undergoing specific learning tasks.

### 3.2. Basal Expression of mPAK3-GFP Proteins in cFos-Positive Cells Impairs Social Recognition Memory

To investigate the functional consequences of basal expression of mPAK3-GFP proteins, we performed various behavioral tests. We first determined that there were no sex-related differences among tetO-sTg, tTA-sTg, or dTg animals in the open field test (Figure S1A–F) or in the three-chamber social interaction test (Figure S1G,H), and therefore, we pooled the data from male and female mice together within each genotype. In addition, there were no differences between tetO-sTg and tTA-sTg animals in the open field test (Figure S2A–F), elevated plus maze (Figure S2G–J), novel object recognition test (Figure S2K–M), contextual (Figure S3A,B) and cued (Figure S3C,D) fear conditioning, and social memory tests (Figure S3E–H), and therefore, unless indicated otherwise, we combined tetO-sTg and tTA-sTg mice into a single control group.

**Figure 3:**
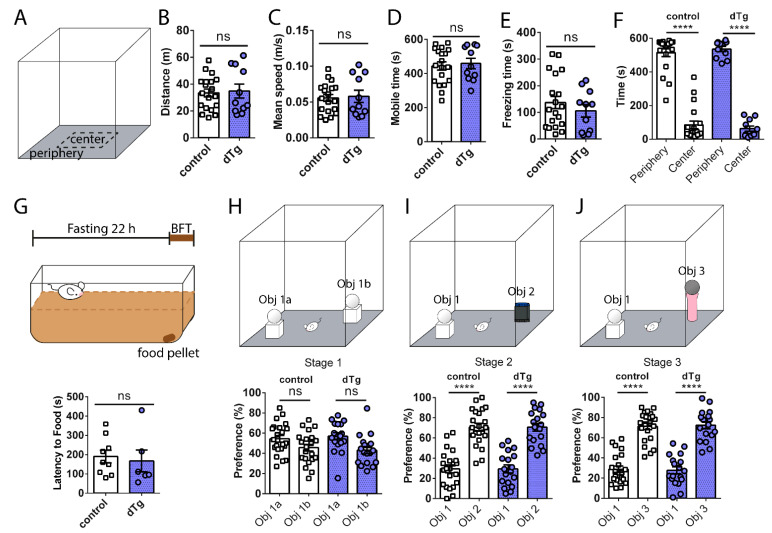
Normal locomotor activity, anxiety, and olfactory behavior in dTg animals. (**A**) Schematic of open field test. No differences between control and dTg mice in (**B**) distance (t_(__28__)_ = 0.2464, *p* = 0.8072), (**C**) mean speed (t_(28)_ = 0.2403, *p* = 0.8118), (**D**) mobile time (t_(28)_ = 0.3778, *p* = 0.7084), (**E**) freezing time (t_(28)_ = 0.8859, *p* = 0.3832), and (**F**) time spent in periphery and center zones (F_(__1,28__)_ = 187.1; *p* < 0.0001, post hoc control *p* < 0.0001, dTg *p* < 0.0001; between genotypes F_(1,28)_ = 0.005496; *p* = 0.9414, post hoc *p* = 0.7674, control *n* = 19, dTg *n* = 11). (**G**) Schematic of buried food test; no differences between genotypes in olfaction (t_(13__)_ = 0.3735, *p* = 0.7148; control *n* = 9, dTg *n* = 6). (**H**–**J** top) Schematic of novel object recognition test. (**H**) No preference during stage 1 of the novel object recognition (F_(1,38)_ = 5.387; *p* = 0.0258, post hoc control *p* = 0.1841, dTg *p* = 0.1244; between genotypes F_(1,38)_ = 41.57; *p* < 0.0001, post hoc *p* = 0.8513; control *n* = 22, dTg *n* = 18). No difference in novelty preference, as determined by (**I**) stage 2 (F_(__1,38__)_ = 56.54; *p* < 0.0001, post hoc control *p* < 0.0001, dTg *p* < 0.0001; between genotypes F_(1,38)_ = 0.1587; *p* = 0.6926, post hoc *p* = 0.9997), and (**J**) stage 3 (F_(__1__,38__)_ = 84.13; *p* < 0.0001, post hoc control *p* < 0.0001, dTg *p* < 0.0001; between genotypes F_(1,38)_ = 2.613; *p* = 0.1143, post hoc *p* = 0.9652), of the novel object recognition. dTg, double transgenic; BFT, buried food test; Obj, object. **** *p* < 0.0001, ns = no significance; two-tailed unpaired *t*-test for two group comparisons and two-way RM ANOVA followed by post hoc Holm–Sidak comparisons for more than two groups. Data are expressed as mean ± SEM. *n* = number of mice.

We examined the basal locomotor and anxiety behavior of dTg mice and found that dTg mice showed similar exploratory behavior as control animals (Figure 3A–E), both with a preference for the periphery zone (Figure 3F). Additionally, dTg animals were able to find a buried food pellet in the buried food test at a similar rate to control animals (Figure 3G). During the novel object recognition test, control and dTg animals both preferred to spend more time with novel objects introduced during stages 2 and 3, compared to the familiar object previously introduced in stage 1 (Figure 3H–J).

**Figure 4:**
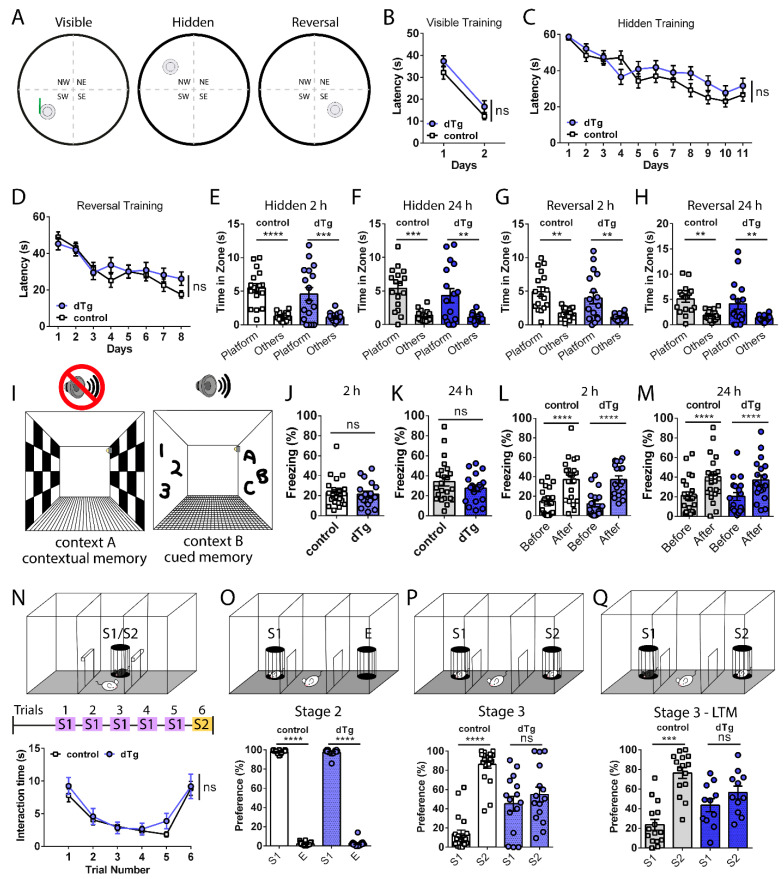
Impaired social recognition memory in three-chamber social tests, but normal spatial and fear learning and memory, in dTg mice. (**A**) Schematics of visible, hidden, and reversal MWM training layout. No differences between control (*n* = 19) and dTg (*n* = 22) mice in escape latency during (**B**) visible platform (F_(1,__39__)_ = 143.7; *p* < 0.0001, post hoc control *p* < 0.0001, dTg *p* < 0.0001; between genotypes F_(1,39)_ = 2.280; *p* = 0.1391), (**C**) hidden platform (between genotypes F_(1,39)_ = 0.9090; *p* = 0.3463), and (**D**) reversal platform (between genotypes F_(1,39)_ = 0.3892; *p* = 0.5364). No differences between control (*n* = 17) and dTg (*n* = 17) mice during the hidden platform probe memory tests at (**E**) 2 h (F_(1,32)_ = 41.77; *p* < 0.0001, post hoc control *p* < 0.0001, dTg *p* = 0.0003; between genotypes F_(1,32)_ = 0.8153; *p* = 0.3733, post hoc platform *p* = 0.4650) and (**F**) 24 h (F_(1,32)_ = 28.65; *p* < 0.0001, post hoc control *p* = 0.0004, dTg *p* = 0.0019; between genotypes F_(1,32)_ = 1.264; *p* = 0.2692, post hoc platform *p* = 0.4308) or reversal platform probe tests at (**G**) 2 h (F_(1,32)_ = 26.16; *p* < 0.0001, post hoc control *p* = 0.0012, dTg *p* = 0.0017; between genotypes F_(1,32)_ = 2.325; *p* = 0.1372, post hoc platform *p* = 0.4064) and (**H**) 24 h (F_(1,32)_ = 19.92; *p* < 0.0001, post hoc control *p* = 0.0038, dTg *p* = 0.0062; between genotypes F_(1,32)_ = 1.709; *p* = 0.2004, post hoc platform *p* = 0.4709). (**I**) Schematics of context A and B used for fear conditioning. (**J**) Short-term 2 h contextual t_(39)_ = 0.3345, *p* = 0.7398, (**K**) long-term 24 h contextual t_(39)_ = 1.135, *p* = 0.2632, (**L**) short-term 2 h cued F_(1,39)_ = 154.8; *p* < 0.0001, post hoc control *p* < 0.0001, dTg *p* < 0.0001; between genotypes F_(1,39)_ = 0.07609; *p* = 0.7841, post hoc before *p* = 0.8592, after *p* = 0.9834, and (**M**) long-term 24 h cued F_(1,39)_ = 46.40; *p* < 0.0001, post hoc control *p* < 0.0001, dTg *p* < 0.0001; between genotypes F_(1,39)_ = 0.04173; *p* = 0.8392, post hoc before *p* = 0.9743, after *p* = 0.9743, fear conditioning memory tests showing no differences between control (*n* = 23) and dTg (*n* = 18) mice. (**N**) Schematic of the five-trial social interaction assay; no differences in social habituation and dishabituation of both groups to sex-matched, juvenile strangers (between genotypes F_(1,45)_ = 1.157; *p* = 0.2878, post hoc trial 1 *p* = 0.8432, trial 5 *p* = 0.6180, trial 6 *p* = 0.9943, control *n* = 29, dTg *n* = 18). (**O**–**Q** top) Schematics of stage 2 and 3 of the three-chamber social interaction test. (**O**) Stage 2 showed similar sociability of control and dTg mice (F(_1,33)_ = 11207; *p* < 0.0001, post hoc control *p* < 0.0001, dTg *p* < 0.0001, between genotypes F_(1,33)_ = 0.01445; *p* = 0.9051, post hoc *p* = 0.7916, control *n* = 20, dTg *n* = 15). (**P**) During stage 3 F_(1,33)_ = 25.67; *p* < 0.0001, post hoc control *p* < 0.0001, dTg *p* = 0.4397, between genotypes F_(1,33)_ = 1.112; *p* = 0.2993, post hoc *p* = 0.0005, dTg mice showed impaired social recognition of a novel S2 compared with S1. (**Q**) Impaired social recognition memory tested 24 h later during stage 3 in dTg mice (F_(1,24)_ = 14.88; *p* = 0.0008, post hoc control *p* = 0.0002, dTg *p* = 0.3293; between genotypes F_(1,24)_ = 0.05879; *p* = 0.8105, post hoc *p* = 0.0459 control *n* = 15, dTg *n* = 11). MWM, Morris Water Maze; dTg, double transgenic; S1, stranger 1; S2, stranger 2; E, empty cage; LTM, long-term memory. ** *p* < 0.01, *** *p* < 0.001, **** *p* < 0.0001, ns = no significance. two-tailed unpaired *t*-test for two group comparisons and two-way RM ANOVA followed by post hoc Holm–Sidak comparisons for more than two groups. Data are expressed as mean ± SEM. *n* = number of mice.

Control and dTg animals were also subjected to three versions of the Morris Water Maze (Figure 4A)—namely, visible, hidden and reversal platform training—to examine their swimming motor skills, simple spatial learning, and flexible spatial learning, respectively. Both groups showed intact swim motor skills and the ability to locate the visible platform (Figure 4B). Then, the animals were trained and eventually able to locate a hidden platform (Figure 4C) and a new platform location during reversal training (Figure 4D). Both groups maintained a short- and long-term memory of the platform location during the hidden and reversal probe tests 2 and 24 h after training (Figure 4E–H). We also subjected control and dTg animals to delayed fear conditioning protocols (Figure 4I) to test their short- and long-term contextual and cued fear memories and found no differences in the levels of freezing between the groups for contextual (Figure 4J,K) or cued memory (Figure 4L,M) when tested 2 h or 24 h after training. Therefore, basal expression of mPAK3-GFP proteins in cFos-positive cells has no significant effect on locomotion, olfaction, novel object recognition, or spatial and fear learning and memory.

We then tested social memory acquisition and retrieval using the five-trial social interaction assay (Figure 4N) and the three-chamber social interaction test (Figure 4O–Q). In the five-trial social interaction assay, where the subject is introduced to a juvenile, sex-matched stranger beginning from the first trial through to the fifth trial, both control and dTg mice showed a gradual habituation and social recognition of familiar stranger S1 (Figure 4N trial 1 vs. 5, control *p* < 0.0001, dTg *p* = 0.0048). Then, introduction to the novel stranger S2 during the final sixth trial led to social dishabituation in both groups, as they displayed increased social interaction with S2 (Figure 4N trial 6, control trial 5 vs. 6 *p* < 0.0001, dTg *p* = 0.0048). Thus, dTg mice behaved normally in the five-trial social interaction task. In another social memory test, the three-chamber social interaction test, when given a choice between S1 or an empty cage (E) during stage 2, both control and dTg mice interacted with S1 more than E (Figure 4O). During stage 3, when a novel S2 was placed into E, control animals preferred interaction with the novel S2 over S1, whereas dTg animals showed similar interaction times between S1 and S2, with no clear preference for the social novelty (Figure 4P). Similar results were seen in a modified three-chamber social interaction test to test long-term memory, in which stage 3—LTM was performed 24 h after stage 2 to test long-term social memory, where dTg showed no preference for S2 (Figure 4Q) despite regular sociability in the prior stage 2, as in stage 2 of short-term memory testing (data not shown). Overall, dTg mice displayed an impairment in a specific form of social recognition memory tested by the three-chamber social interaction test.

### 3.3. Administration of DOX Reverses Behavioral Deficits Seen in dTg Animals

**Figure 5:**
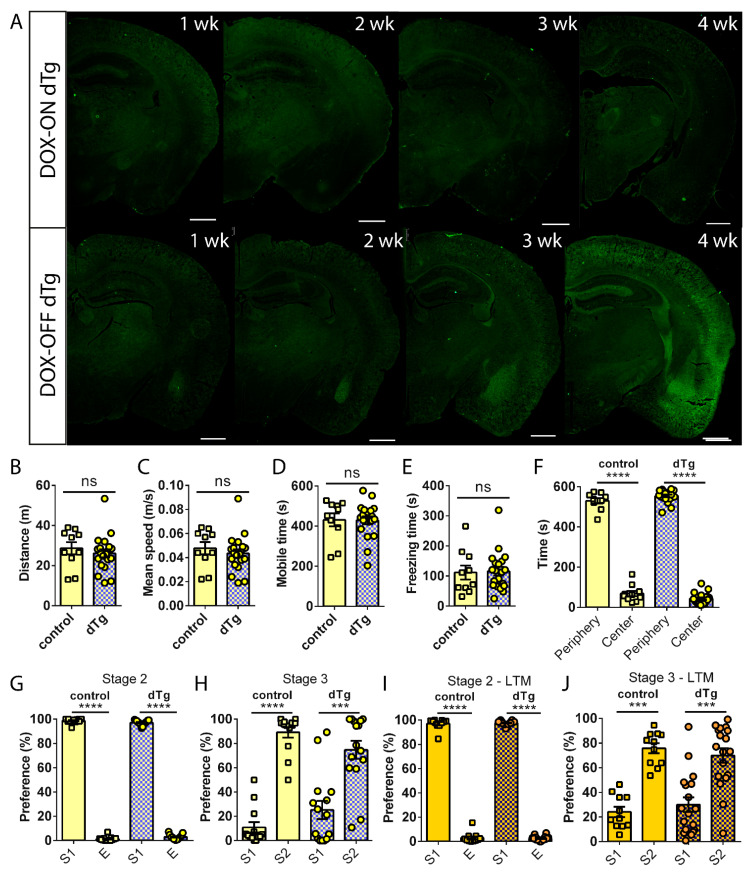
DOX administration rescues social recognition deficits in dTg animals without affecting basal locomotor activity. (**A**) The global expression of mPAK3-GFP proteins in dorsal coronal dTg half brain sections after the gradual administration and removal of DOX. Scale bar = 1000 μm. (**B**–**F**) The locomotor and anxiety behavior of control and dTg mice during the open field test showing no significant differences after 4 weeks of DOX administration. No differences in (**B**) distance t_(28)_ = 0.7009, *p* = 0.4891; (**C**) mean speed t_(28)_ = 0.7231, *p* = 0.4756; (**D**) mobile time t_(28)_ = 0.06681, *p* = 0.9472; (**E**) freezing time t_(28)_ = 0.1653, *p* = 0.8699; and (**F**) time spent in periphery and center zones F_(1,28)_ = 1379; *p* < 0.0001, post hoc control *p* < 0.0001, dTg *p* < 0.0001; between genotypes F_(1,28)_ = 1.130; *p* = 0.2969, post hoc *p* = 0.1112) of the open field test. (**G**) Sociability was unaffected during stage 2 F_(1,26)_ = 15911; *p* < 0.0001, post hoc control *p* < 0.0001, dTg *p* < 0.0001; between genotypes F_(1,26)_ = 1.518; *p* = 0.2289, post hoc *p* = 0.0777, of the three-chamber social interaction test while rescuing basal short-term social recognition deficit during (**H**) stage 3 F_(1,26)_ = 50.31; *p* < 0.0001, post hoc control *p* < 0.0001, dTg *p* = 0.0004; between genotypes F_(1,26)_ = 0.1108; *p* = 0.7419, post hoc *p* = 0.2216, in dTg mice after 4 weeks of DOX administration; control *n* = 13, dTg *n* = 15. (**I**) Similar sociability in stage 2 LTM: F_(1,27)_ = 6610; *p* < 0.0001, post hoc control *p* < 0.0001, dTg *p* < 0.0001; between genotypes F_(1,27)_ = 3.379; *p* = 0.0770, post hoc *p* = 0.8692, and (**J**) social recognition rescue in stage 3 LTM: F_(1,27)_ = 30.66; *p* < 0.0001, post hoc control *p* = 0.0010, dTg *p* = 0.0010; between genotypes F_(1,27)_ = 0.7792; *p* = 0.3852, post hoc *p* = 0.7263; control *n* = 11, dTg *n* = 18). dTg, double transgenic; DOX, doxycycline; DOX-ON, on doxycycline; DOX-OFF, off doxycycline; S1, stranger 1; S2, stranger 2; E, empty cage; LTM, long-term memory. *** *p* < 0.001, **** *p* < 0.0001, ns = no significance; two-tailed unpaired *t*-test for two group comparisons and two-way RM ANOVA followed by post hoc Holm–Sidak comparisons for more than two groups. Data are expressed as mean ± SEM. *n* = number of mice.

To test the validity of the tetracycline-inducible system in dTg mice, we administered DOX to our mice for a total of 4 weeks and examined the mPAK3-GFP protein expression in dorsal coronal brain sections of dTg mice at weekly intervals. We visualized fewer mPAK3-GFP proteins in the cortex, HPC, and BLA with increased duration of DOX administration, and mPAK3-GFP levels resembled those of control animals under basal conditions after 3 weeks (Figure 5A, DOX-ON row). We also performed reverse experiments by examining the mPAK3-GFP expression levels after removing DOX and found that more mPAK3-GFP proteins appeared in the cortex, HPC, and BLA of dTg sections as the time interval increased after DOX removal, with levels similar to basal dTg expression returning at 4 weeks after removal (Figure 5A, DOX-OFF row).

To determine the effect of turning off mPAK3-GFP expression on behavior, we tested the locomotor and anxiety behavior of control and dTg animals in the open field test after 4 weeks of DOX administration. We found that the exploratory behavior of control and dTg animals was similar (Figure 5B–E), again with both groups preferring to spend time in the periphery zone rather than in the center (Figure 5F). We also looked at the effects of DOX on short-term social memory of the three-chamber social interaction test, and after 4 weeks of DOX, both control and dTg animals preferred to interact with S1 over E in stage 2 (Figure 5G) and showed a preference for the novel S2 over S1 in stage 3 (Figure 5H), similar to the social preference seen in control animals under basal conditions. Similar results were observed in the three-chamber social interaction long-term memory test (Figure 5I,J). Therefore, turning off transgenic mPAK3-GFP expression through DOX administration rescued social memory deficits in dTg mice.

### 3.4. dTg Animals Can Be Used for a Social Pairing Protocol to Label cFos-Expressing Cells

**Figure 6:**
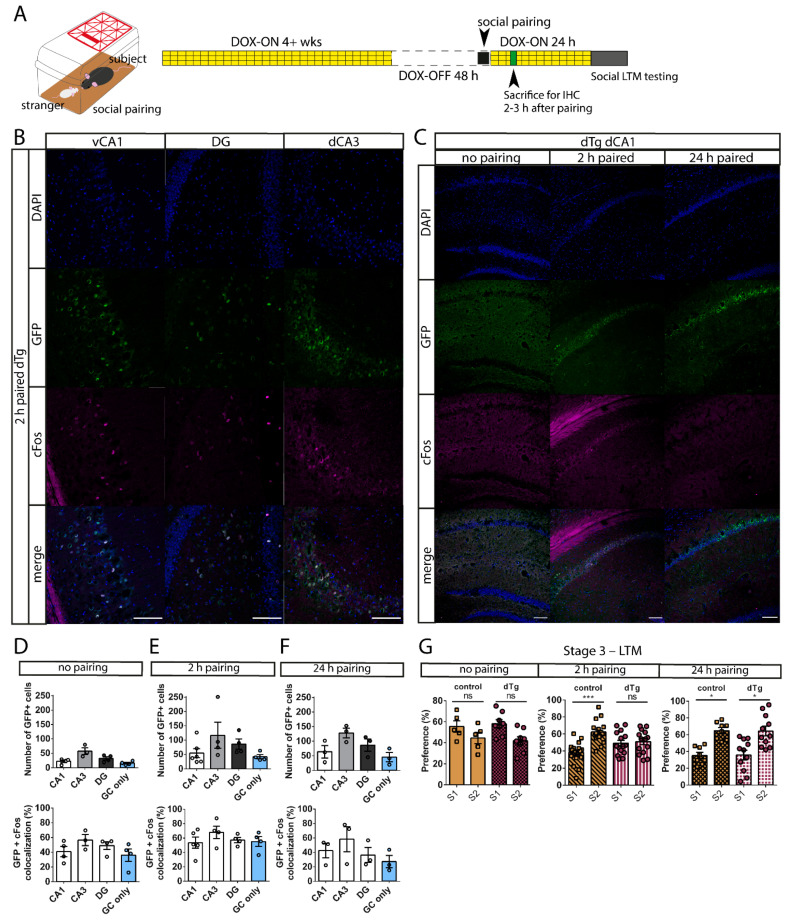
Social pairing-induced expression of mPAK3-GFP proteins impairs social recognition memory. (**A**) Schematics showing home-cage social pairing and DOX administration and removal to induce expression of mPAK3-GFP proteins. (**B**) Sample immunostaining images showing the expression of mPAK3-GFP proteins and overlapping Fos protein expression in the vCA1, DG, and dCA3 of dTg mice after a 2 h social pairing interaction during the DOX-OFF window. Scale bars: 100 μm. (**C**) Sample immunostaining images showing the effects of different pairing durations during the DOX-OFF window on the mPAK3-GFP expression in the dCA1 of dTg mice. Scale bars: 100 μm. (**D**–**F**) Summary graphs showing the number of GFP-positive cells and percentage of GFP and Fos overlap over total GFP-positive cells in various hippocampal regions; (**D**) not paired CA1 *n* = 4, CA3 *n* = 3, DG *n* = 4; (**E**) 2 h paired CA1 *n* = 5, CA3 *n* = 4, DG *n* = 4; (**F**) 24 h paired CA1, CA3, DG *n* = 3. (**G**) A 2 h but not a 24 h social pairing interaction resulting in a social recognition deficit during stage 3 of the three-chamber social interaction test (no pairing: F_(1,11)_ = 4.511; *p* = 0.0572, post hoc control *p* = 0.2968, dTg *p* = 0.1280; between genotypes F_(1,11)_ = 0.6044; *p* = 0.4533, post hoc *p* = 0.9033; control *n* = 5, dTg *n* = 8; 2 h pairing: F_(1,33)_ = 8.714; *p* = 0.0058, post hoc control *p* = 0.0004, dTg *p* = 0.7812; between genotypes F_(1,33)_ = 0.04172; *p* = 0.8394, post hoc *p* = 0.0275; control *n* = 20, dTg *n* = 15; 24 h pairing: F_(1,17)_ = 15.17; *p* = 0.0012, post hoc control *p* = 0.0185, dTg *p* = 0.0179; between genotypes F_(1,17)_ = 0.1398; *p* = 0.7131, post hoc *p* = 0.9972; control *n* = 8, dTg *n* = 11). dCA1, dorsal CA1; dCA3, dorsal CA3; vCA1, ventral CA1; DG, dentate gyrus; GC, granule cell of dentate gyrus; DOX-ON, on doxycycline; DOX-OFF, off doxycycline; S1, stranger 1; S2, stranger 2; LTM, long-term memory; dTg, double transgenic. * *p* < 0.05, *** *p* < 0.001, ns = no significance; two-tailed unpaired *t*-test for two group comparisons and two-way RM ANOVA followed by post hoc Holm–Sidak comparisons for more than two groups. Data are expressed as mean ± SEM. *n* = number of mice.

Since our tetracycline-inducible system offers reversible expression of the mPAK3-GFP transgene, we optimized the system to label cells activated during acquisition of a memory for social identity. We first placed the mice on DOX for 4 weeks to turn off transgenic gene expression and then allotted for a short 48 h window where DOX was removed (DOX-OFF) to allow the expression of the transgene to return during a home-cage social interaction (social pairing) with a juvenile, sex-matched stranger (Figure 6A). After this 48 h window, subjects were immediately placed back on DOX to prevent further transgene expression, where the subjects were either sacrificed for immunohistochemistry 2–3 h after the pairing (Figure 6B–F) or tested for social memory 24 h after the pairing (Figure 6G). We found that dTg animals showed mPAK3-GFP protein expression in the dorsal and ventral hippocampal CA1 (54.24 ± 16.04 cells), DG (86.00 ± 17.68 cells), and dorsal CA3 regions (116.8 ± 45.73 cells) (Figure 6B,C), as well as in L4/5 of the somatosensory cortex and the superficial layers of the lateral entorhinal cortex (LEC) (Figure S4A), after a 2 h social pairing interaction. We also subjected the mice to different durations of social pairing interaction to examine the differences in transgenic protein expression and behavior, and confirmed that without a pairing session, dTg animals showed lower (22.25 ± 4.535 cells, 40.90 ± 6.878% Fos overlap) mPAK3-GFP protein expression (Figure 6D), whereas 2 h (53.60 ± 7.665% overlap with Fos) and 24 h pairing sessions showed mPAK3-GFP expression in the dorsal CA1 region (24 h paired CA1 63.92 ± 21.31 cells, 42.50 ± 10.11% Fos overlap) (Figure 6E,F). In addition, control and dTg subjects who were not paired with a stranger showed no preference for either stranger in the subsequent social memory test (Figure 6G, no pairing). In contrast, dTg subjects who were paired for 2 h showed impaired social recognition for S2, but control subjects did not show the same deficit (Figure 6G, 2 h pairing). Finally, when control and dTg subjects were social paired for 24 h, they both showed a preference for a novel S2 during the social memory test 24 h after the end of pairing (Figure 6G, 24 h pairing). In summary, the induced expression of mPAK3-GFP proteins in cFos-positive cells, after a 2 h home-cage social pairing interaction, was sufficient to impair social recognition memory in dTg mice.

## 4. Discussion

Our basal immunohistochemistry results suggest that dTg animals express mPAK3-GFP proteins in cFos-expressing cells, more in excitatory than inhibitory neurons, of regions that are implicated in learning and memory, such as the LEC, HPC, particularly the CA1 and CA3 regions, and retrosplenial and sensory cortices, confirming the role of PAK3 in learning and memory. Fewer mPAK3-GFP cells were identified in granule cells (GCs) of the DG, in accordance with previous studies suggesting that GCs are responsible for specific memory episodes and are not easily triggered [46,47]. Although most mPAK3-GFP cells colocalized with cFos proteins, the lack of complete colocalization may be due to the delayed degradation of mPAK3-GFP proteins as opposed to Fos proteins, which inherently degrade within hours of expression [37]. Our immunostaining results also showed a low level of mPAK3-GFP expression in tTA-sTg or tetO-sTg animals under basal conditions, and this expression may be due to the short-lived EGFP protein fused to the Fos-tTA transgene [39,40] or leaky expression of the mPAK3-GFP transgene. Previous studies have indicated that tetracycline-inducible systems are susceptible to varied levels of leaky expression [48,49]. Nevertheless, we found that the leaky expression in our control animals was minimal and did not impair their behavior or memory.

Although we found mPAK3-GFP proteins expressed in several brain regions, including the LEC to HPC pathway, we detected no effects on the basal behavior of dTg mice, such as anxiety and exploratory, olfactory, and novelty preference, as dTg animals showed no changes in open field, buried food, and novel object recognition tests, respectively. In addition, we did not observe a deficit in spatial or fear memory acquisition or recall as evidenced by our Morris Water Maze and fear conditioning results. Our spatial memory test supports the results of a recent study that demonstrated that mPAK3 protein selectively impaired strained, massed spatial memory training protocols but not distributed spatial training such as our training protocol [15]. On the other hand, fear memory acquisition and retrieval may also be compensated by alternative pathways due to its evolutionary importance for survival [50]. Our dTg mice were impaired in a social recognition task when subjected to a choice between familiar and novel strangers in the three-chamber social interaction test, but interestingly, the dTg mice were able to form a social memory of the familiar stranger during the five-trial social interaction test. The specificity of the social memory deficit suggests that PAK3 proteins may specifically be involved in situations where subjects are allowed to interact with strangers of their choice in a common context. Alternatively, if subjects are repeatedly introduced to a single stranger over time, as in the five-trial social interaction assay, subjects seemed to develop a social memory for S1. Our social results coincide with our spatial memory findings, further suggesting a specific role of PAK signaling in condensed training, and deficits in such may be overcome if training is repeatedly given.

The tetracycline-inducible system in our mouse model was also validated, as DOX rescued the social deficits that were present in dTg mice under basal conditions, demonstrating the reversibility of mPAK3 transgenic expression. This allowed us to test a social interaction pairing protocol to isolate the expression of mPAK3-GFP proteins to a single 2 h social interaction between dTg subjects and a juvenile, sex-matched stranger. Importantly, this social pairing induced expression of mPAK3-GFP proteins that resulted in subsequent social recognition deficits similar to those observed under basal conditions. Using this pairing protocol, we identified a subset of cFos- and mPAK3-GFP-expressing cells in the dorsal and ventral CA1, CA3, and DG, activated by the social pairing event. These results highlight the importance of PAK signaling in this subset of cFos-positive cells, which corroborate previous studies identifying regions important for memory acquisition [51,52]. The number of activated cells appeared to increase with the duration of social pairing, as subjects who were not paired had fewer mPAK3-GFP-positive cells, likely a result of background activation, whereas 2 and 24 h paired mice had similar levels of mPAK3-GFP cells. Interestingly, mPAK3-GFP-expressing cells of both 2 and 24 h paired mice overlapped with Fos-expression approximately half the amount of time, suggesting some mPAK3 proteins were previously expressed and no longer active, allowing for a potential temporal distribution within the same region. It is interesting to note that dTg animals socially paired for 24 h did not show any social recognition deficits, as opposed to those with a 2 h pairing session. This could be attributed to the social memory being acquired under the residual effects of DOX or because the social pairing interaction overcame the initial social deficit; however, further experiments are needed to address these possibilities. Nevertheless, our results indicate that disruption of PAK signaling in a subset of cFos-positive cells specifically activated during social interaction is sufficient to impair social recognition memory, underscoring the critical importance of this signaling pathway in social behavior.

An unresolved question is whether the activation of these cFos positive cells in vivo, specifically during social pairing, is able to alter or enhance social memory. This could be addressed by combining the transgenic mice with an optogenetic method to manipulate these cells acutely. In addition, how mPAK3-GFP proteins in cFos-positive cells affect social memory is yet to be investigated. It is possible that PAK signaling affects neuronal activation through synaptic and spine regulation by interacting with cAMP response element binding (CREB) proteins and actin-binding regulator, cofilin [13,17]. It would be important to examine synaptic function, spine morphology, and CREB/cofilin expression in these cFos-positive cells in the future. An additional limitation of our study is the inherent leaky expression present in transgenic model systems [48,49]. Although the minimal background transgenic expression did not affect the behaviors of our animal groups, future studies must acknowledge this shortcoming when comparing mPAK3-GFP expression levels between animals. Furthermore, the cessation and re-expression of transgenic mPAK3 proteins upon addition or removal of DOX are not immediate. Therefore, modifications to our social pairing protocol would need to take the delayed action of DOX into consideration. As PAK signaling is also associated with many brain disorders, investigating specific roles of PAKs in engram cells is not only important for the understanding of molecular mechanisms of memory formation but also important for the treatment of related brain diseases.

## 5. Conclusions

In the present study, we present a novel mouse model that can be used to study the role of PAK3 proteins in social engram cells. We demonstrated that the expression of mPAK3-GFP proteins during a single 2 h social interaction can be visualized and impairs social recognition memory. Since PAK signaling plays important roles in synaptic plasticity and is implicated in various brain diseases, our animal model provides a valuable tool for studying the molecular mechanisms underlying brain function and dysfunction related to PAK.

## Data Availability

All the supporting data are presented in this paper or available online at www.mdpi.com (accessed on 31 October 2021).

## Supplementary Materials

The following are available online at https://www.mdpi.com/article/10.3390/cells10113010/s1, Figure S1: No sex differences observed between tetO-sTg, tTA-sTg and dTg animals in basal locomotor and anxiety levels, or social memory, Figure S2: No genotype differences observed between tetO-sTg and tTA-sTg animals in basal locomotion, anxiety, or novelty preference, Figure S3: No genotype differences observed between tetO-sTg and tTA-sTg animals in basal fear and social memory, Figure S4: Additional mPAK3-GFP cells are activated in the cortex and LEC after a 2-h social pairing session.
