# Supplementary material for: Disruption of PAK3 Signaling in Social Interaction Induced cFos Positive Cells Impairs Social Recognition Memory
Source: Cells. 2021 Nov 4;10(11):3010. doi: 10.3390/cells10113010 (PMC8616103; doi:10.3390/cells10113010)
Supplement: Supplementary file 1 [file cells-10-03010-s001.zip › cells-1413499-SI.pdf]

Supplementary Figures

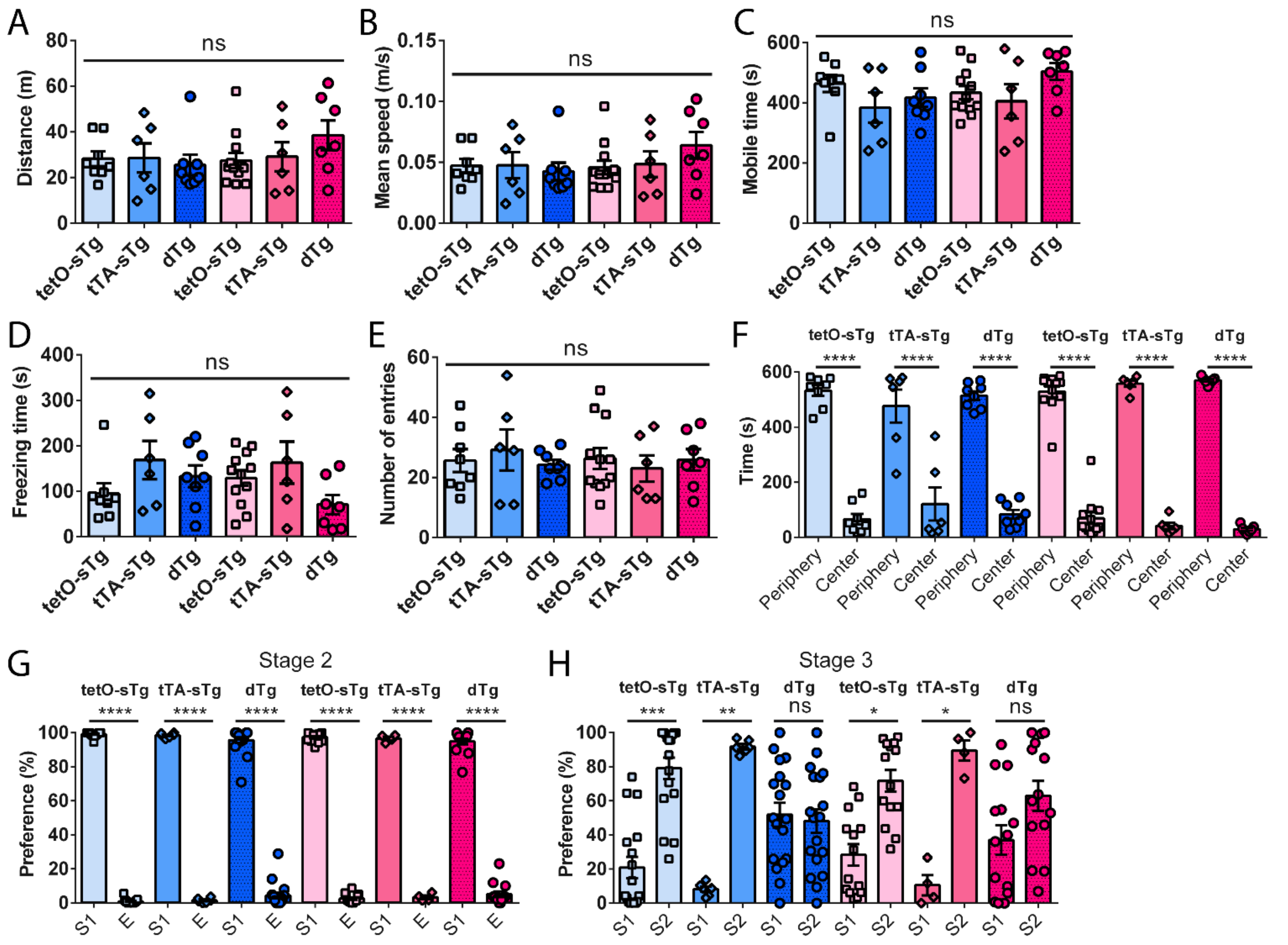

Figure S1. No sex differences observed between tetO-sTg, tTA-sTg and dTg animals in basal locomotor and anxiety levels, or social memory. Male and female tetO-sTg, tTA-sTg, and dTg mice showed no differences in (A) distance F (5, 41) = 0.8455, p = 0.5256, (B) mean speed F (5, 41) = 0.8337, p = 0.5334, (C) mobile time F (5, 41) = 1.414, p = 0.2397, (D) freezing time F (5, 41) = 1.685, p = 0.1599, (E) entries to the center F (5, 41) = 0.2249, p = 0.9497, and (F) time spent in periphery and center zones F (1, 41) = 474.1; p < 0.0001, post-hoc all groups p < 0.0001, between groups F (5, 41) = 0.3012; p = 0.9093, of the open field test; male tetO-sTg n = 8, male tTA-sTg n = 6, male dTg n = 8, female tetO-sTg n = 12, female tTA-sTg n = 6, female dTg n = 7. Males and females of each control and dTg mouse group also showed no differences in (G) stage 2 F (1, 67) = 5283; p < 0.0001, post-hoc all groups p < 0.0001, between groups F (5, 67) = 0.5788; p = 0.7160, or (H) stage 3 F (1, 67) = 43.12; p < 0.0001, post-hoc analysis: male tetO-sTg p = 0.0002, male tTA-sTg p = 0.0013, male dTg p = 0.7670, female tetO-sTg p = 0.0128, female tTA-sTg p =0.0128, female dTg p = 0.1403, between groups F (5, 67) = 2.303; p = 0.0543, post-hoc male tetO vs. dTg p = 0.0210, male tTA vs. dTg p = 0.0196, of the three-chamber social interaction test; male tetO-sTg n = 17, male tTA-sTg n = 6, male dTg n = 18, female tetO-sTg n = 14, female tTA-sTg n = 4, female dTg n = 14. dTg, double transgenic; sTg, single transgenic; S1, stranger 1; S2, stranger 2; E, empty cage. *p < 0.05, **p < 0.01, ***p < 0.001, **** p <

0.0001, two-tailed unpaired t-test, two-way RM ANOVA, post-hoc Holm-Sidak comparisons. Data are expressed as mean ± SEM. n = number of mice.

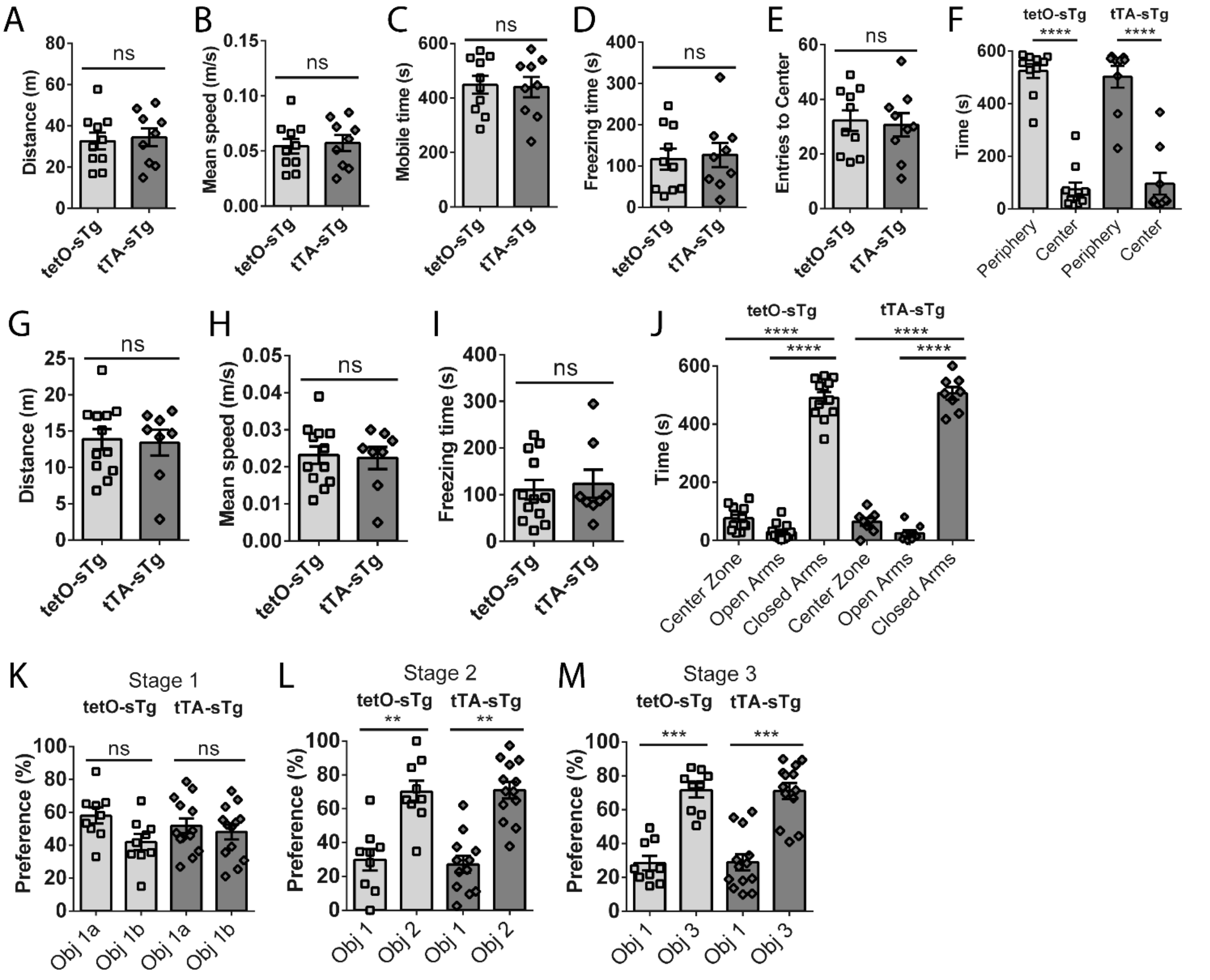

Figure S2. No genotype differences observed between tetO-sTg and tTA-sTg animals in basal locomotion, anxiety, or novelty preference. The tetO-sTg and tTA-sTg mouse groups showed no differences in (A) distance t (17) = 0.2980, p = 0.7693, (B) mean speed t (17) = 0.2856, p = 0.7786, (C) mobile time t (17) = 0.1703, p = 0.8668, (D) freezing time t (17) = 0.2743, p = 0.7872, (E) entries to the center t (17) = 0.2687, p = 0.7914, and (F) time spent in periphery and center zones F (1, 17) = 80.28; p < 0.0001, post-hoc both groups p < 0.0001, between genotypes F (1, 17) = 0.2974; p = 0.5926, of the open field test; tetO-sTg n = 10, tTA-sTg n = 9. TetO-sTg and tTA-sTg mouse groups also showed no differences in (G) distance t (18) = 0.2007, p = 0.8432, (H) mean speed t (18) = 0.2102, p = 0.8359, (I) freezing time t (18) = 0.3654, p = 0.7190, and (J) time spent in arms F (2, 36) = 392.0; p < 0.0001, post-hoc center-closed and open-closed arms of both groups p < 0.0001, between genotypes F (1, 18) = 0.02684; p = 0.8717, post-hoc p = 0.8480, of the elevated plus maze test; tetO-sTg n = 12, tTA-sTg n = 8. No differences were found in (K) stage 1 F (1, 20) = 2.151; p = 0.1580, post-hoc tetO-sTg p = 0.2552, tTA-sTg p = 0.6710, between genotypes F (1, 20) = 1.176; p = 0.2910, post-hoc p = 0.5975, (L) stage 2 F (1, 20) = 26.90; p < 0.0001, post-hoc tetO-sTg p = 0.0035, tTA-sTg p = 0.0010, between genotypes F (1, 20) = 0.02525; p = 0.8753, post-hoc p = 0.9929, or (M) stage 3 F (1, 20) =

41.36; $p < 0.0001$, post-hoc tetO-sTg $p = 0.0004$, tTA-sTg $p = 0.0001$, between genotypes $F (1, 20) = 33.68$; $p < 0.0001$, post-hoc $p = 0.9973$, of tetO-sTg and tTA-sTg groups for the novel object recognition test; tetO-sTg n = 9, tTA-sTg n = 13. sTg, single transgenic; Obj, object. **p < 0.01, ***p < 0.001, **** p < 0.0001, two-tailed unpaired t-test, two-way RM ANOVA, post-hoc Holm-Sidak comparisons. Data are expressed as mean ± SEM. n = number of mice.

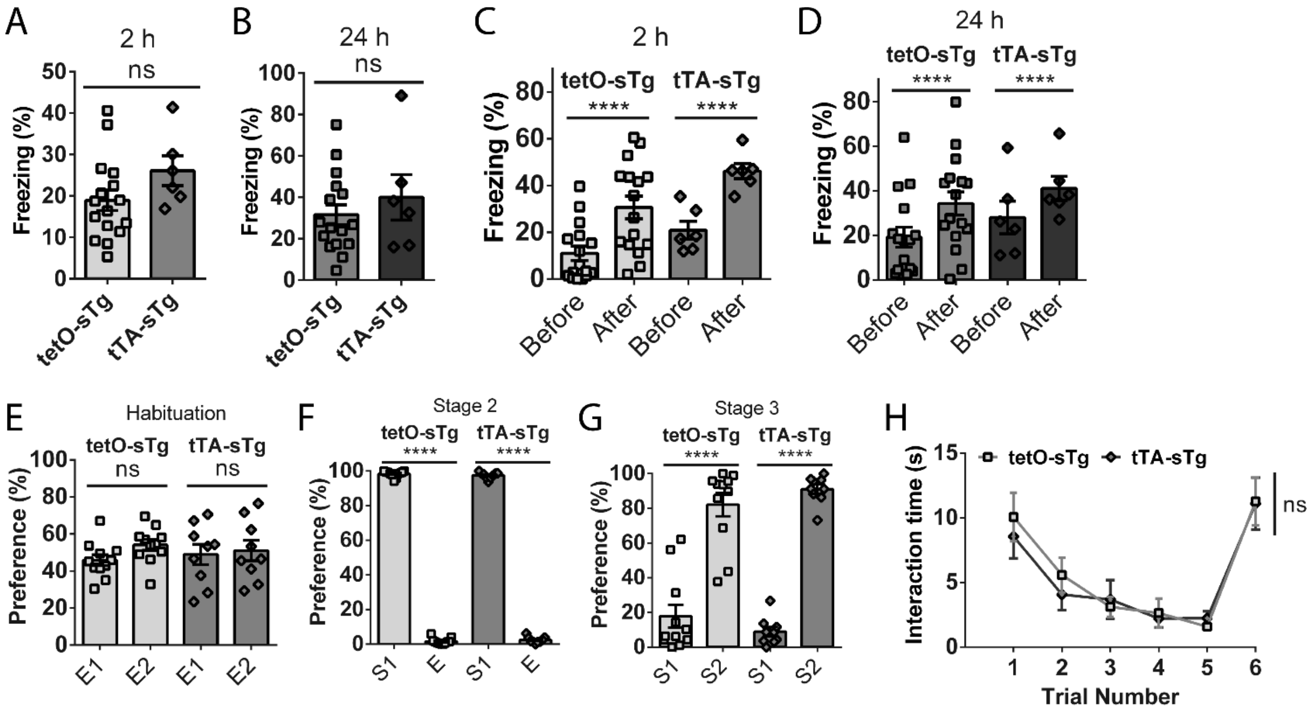

Figure S3. No genotype differences observed between tetO-sTg and tTA-sTg animals in basal fear and social memory. (A) Short-term contextual t (20) = 1.552, p = 0.1365, (B) long-term contextual t (20) = 0.8181; p = 0.4229, (C) short-term cued F (1, 20) = 67.59; p < 0.0001, post-hoc tetO-sTg p < 0.0001, tTA-sTg p < 0.0001, between genotypes F (1, 20) = 3.877; p = 0.0630, post-hoc before p = 0.1668, after p = 0.0635, and (D) long-term cued F (1, 20) = 24.36; p < 0.0001, post-hoc tetO-sTg p = 0.0001, tTA-sTg p = 0.0142, between genotypes F (1, 20) = 0.8522; p = 0.3669, post-hoc before p = 0.5498, after p = 0.5498, fear conditioning memory tests also showed no differences between tetO- and tTA-sTg mice; tetO-sTg n = 16, tTA-sTg n = 6. No differences between tetO- and tTA-sTg animals during (E) habituation (stage 1) F (1, 18) = 0.7699; p = 0.3918, tetO-sTg p = 0.5232, tTA-sTg p = 0.8152, between genotypes F (1, 18) = 17.16; p = 0.0006, post-hoc p = 0.8419, (F) stage 2 F (1, 18) = 13449; p < 0.0001, post-hoc tetO-sTg p < 0.0001, tTA-sTg p < 0.0001, between genotypes F (1, 18) = 0.2882; p = 0.5979, post-hoc p = 0.5496, or (G) stage 3 F (1, 18) = 87.68; p < 0.0001, post-hoc tetO-sTg p < 0.0001, tTA-sTg p < 0.0001, between genotypes F (1, 18) = 2.889; p = 0.1064, post-hoc p = 0.4556, of the three-chamber social interaction test; tetO-sTg n = 11, tTA-sTg n = 9. (H) The five-trial social interaction assay showed no differences in social habituation and dishabituation of both control groups to sex-matched, juvenile strangers; F (5, 100) = 16.02; p < 0.0001, post-hoc tetO-sTg trial 1 vs. 5 p < 0.0001, tetO-sTg trial 5 vs. 6 p < 0.0001, tTA-sTg trial 1 vs. 5 p = 0.0328, tTA-sTg trial 5 vs. 6 p = 0.0007, between genotypes F (1, 20) = 0.1944; p = 0.6640, post-hoc trial 1 p = 0.9707, trial 5 p = 0.9953, trial 6 p = 0.9953, tetO-sTg n = 13, tTA-sTg n = 9. dTg, double transgenic; sTg, single transgenic; S1, stranger 1; S2, stranger 2; E, empty cage. **** p < 0.0001, two-tailed unpaired t-test, two-way RM ANOVA, post-hoc Holm-Sidak comparisons. Data are expressed as mean ± SEM. n = number of mice.

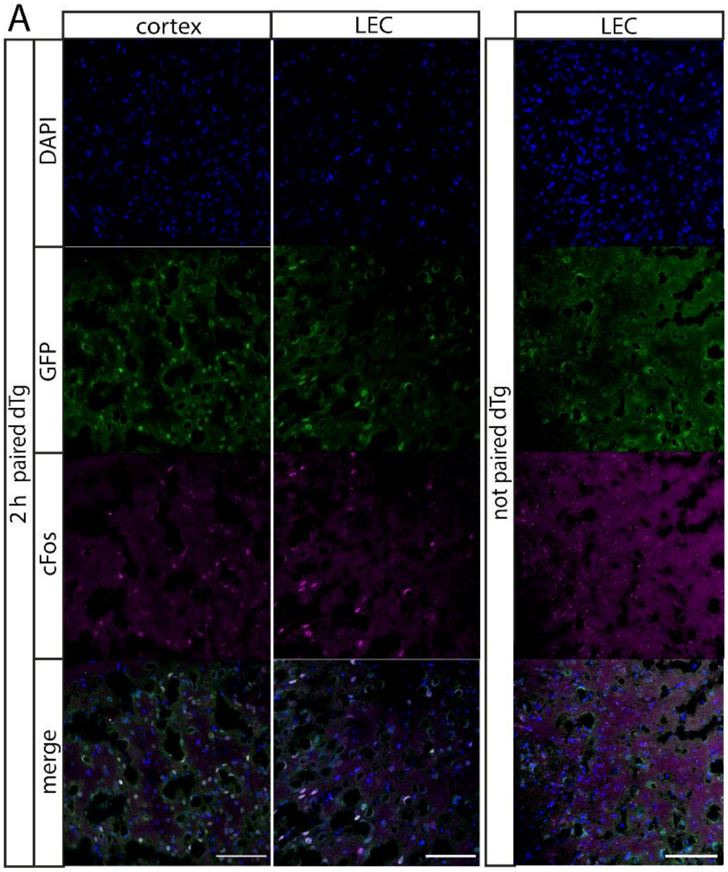

Figure S4. Additional mPAK3-GFP cells are activated in the cortex and LEC after a 2-hour social pairing session. (A) The expression of mPAK3-GFP proteins and overlapping cFos protein expression in L4/5 of the somatosensory cortex (cortex) and LEC of dTg and tTA-sTg mice after a 2 h social pairing interaction. Scale bars: 100 µm. dTg, double transgenic; sTg, single transgenic; LEC, lateral entorhinal cortex.
